# Exon First Nucleotide Mutations in Splicing: Evaluation of *In Silico* Prediction Tools

**DOI:** 10.1371/journal.pone.0089570

**Published:** 2014-02-21

**Authors:** Lucie Grodecká, Pavla Lockerová, Barbora Ravčuková, Emanuele Buratti, Francisco E. Baralle, Ladislav Dušek, Tomáš Freiberger

**Affiliations:** 1 Molecular Genetics Laboratory, Centre for Cardiovascular Surgery and Transplantation, Brno, Czech Republic; 2 Central European Institute of Technology, Masaryk University, Brno, Czech Republic; 3 International Centre for Genetic Engineering and Biotechnology, Trieste, Italy; 4 Institute of Biostatistics and Analyses, Masaryk University, Brno, Czech Republic; 5 Institute of Clinical Immunology and Allergology, St. Anne’s University Hospital and Masaryk University, Brno, Czech Republic; University of Crete, Greece

## Abstract

Mutations in the first nucleotide of exons (E^+1^) mostly affect pre-mRNA splicing when found in AG-dependent 3′ splice sites, whereas AG-independent splice sites are more resistant. The AG-dependency, however, may be difficult to assess just from primary sequence data as it depends on the quality of the polypyrimidine tract. For this reason, *in silico* prediction tools are commonly used to score 3′ splice sites. In this study, we have assessed the ability of sequence features and *in silico* prediction tools to discriminate between the splicing-affecting and non-affecting E^+1^ variants. For this purpose, we newly tested 16 substitutions *in vitro* and derived other variants from literature. Surprisingly, we found that in the presence of the substituting nucleotide, the quality of the polypyrimidine tract alone was not conclusive about its splicing fate. Rather, it was the identity of the substituting nucleotide that markedly influenced it. Among the computational tools tested, the best performance was achieved using the Maximum Entropy Model and Position-Specific Scoring Matrix. As a result of this study, we have now established preliminary discriminative cut-off values showing sensitivity up to 95% and specificity up to 90%. This is expected to improve our ability to detect splicing-affecting variants in a clinical genetic setting.

## Introduction

The generation of functional mRNA from a primary transcript requires the precise removal of introns and the ligation of adjacent exons. Splicing accuracy is ensured by the specific interactions of *trans-*splicing factors with *cis-*splicing sequence elements, the splice sites, branch site (BS) and additional regulatory elements, splicing enhancers and silencers. These sequences tend to be highly degenerate with the exception of the almost invariant dinucleotides at the exon-intron boundaries. Variations in the highly conserved “AG” dinucleotide in the acceptor splice site (3′ss) generally disrupt splicing of the affected gene. However, changes elsewhere in the 3′ss have consequences that are not as obvious [1], [2]. In particular, mutations in the first nucleotide of exons (E^+1^) lead to various splicing outcomes that are influenced by the AG-dependence of the affected splice site [3]. It has been demonstrated that variations in the E^+1^ position in an AG-dependent 3′ss predispose the mRNA to aberrant splicing, whereas this process is not affected when the variation is located in an AG-independent acceptor site [3]. The AG-dependence is a feature of introns that requires an intact AG dinucleotide already for the first step of the splicing reaction, whereas AG-independent introns require the AG only in the second transesterification step [4]. In general, AG-dependent introns contain shorter and more degenerate polypyrimidine tracts (PPT) and require the binding of both subunits of U2 snRNP auxiliary factor (U2AF65 and U2AF35) for the first transesterification step to occur, whereas AG-independent introns contain more consensual and longer PPTs located closer to the splice site and do not rely on U2AF35 binding [5], [6].

Fu et al. [3] have recently observed that the length of the longest pyrimidine stretch (PPS) in the PPT represents the best differentiating feature for intron AG-dependence; however, it is still difficult to determine the AG-dependence by simply examining the DNA sequence (and especially the potential splicing affection represented by an E^+1^ mutation). Currently, several computational instruments are available that can predict the location of the splice site and its strength in terms of score (resemblance to an ideal consensual sequence) as well as the location and strength of the BS, PPT, and other splicing regulatory elements (SREs). All these factors, when correctly integrated, might make the determination of intron AG-dependence easier. Although the reliability of *in silico* tools is still limited and differs significantly between various algorithms (results often require experimental confirmation) computational predictions still represent an important starting tool when prioritizing an unclassified variant for functional validation [2].

Originally, prediction tools predicted splice site quality based on nucleotide frequencies of independent positions (e.g. Shapiro and Senepathy matrix) [7]. Following this initial approach, more sophisticated predictive strategies were developed such as machine-learning (used in Neural Network Splice Site Prediction Tool, NNSplice) and the maximum entropy model (used in Maximum Entropy Based Scoring Method, MaxEnt) [8], [9]. The machine-learning approach recognizes sequence patterns upon training with authentic splice sites sequences [8]. On the other hand, the maximum entropy model approximates short-sequence motifs distribution accounting for nonadjacent as well as adjacent dependencies between positions of the splice site [9].

In parallel to these approaches dedicated at scoring acceptor and donor sites there has also been the appearance of additional strategies aimed at predicting the other basic splicing consensus sequences. For example the existing predictions of BS are based on a position-specific weight matrix, whilst PPT presence and quality is predicted using algorithms that account for length and pyrimidine content [10], [11]. Finally, predictions for the presence of SRE elements have also made their appearance. In general, SRE prediction programs were basically built up in two ways. Programs of the first type are based on looking for the presence of SRE motifs derived from statistical analyses of differences in oligomer frequencies between various sequences (such as exons vs. introns, noncoding exons vs. pseudo exons, etc.) [12], [13]. On the other hand, the second type of programs is composed by SRE prediction algorithms based on *in vitro* and *in cellulo* experimental approaches such as SELEX or splicing reporter system experiments [14], [15].

At the moment, no prediction program can be said to fully predict consensus and SRE sequences with 100% accuracy and their reliability seems to differ from system to system. Nonetheless, all studies performed so far agree that using a combination of all these programs represents the best strategy to predict possible splicing outcomes.

In this study, we analyzed *in vitro* the splicing potential of sixteen E^+1^ mutations in genes related to the development of human hereditary disorders and tested the efficiency of several freely available *in silico* instruments to predict the splicing outcome of this mutation type. Being the first report evaluating the performance of *in silico* tools to predict the outcome of E^+1^ mutations we believe that these data could serve as preliminary guidance for genetic diagnostics and might also be helpful to bioinformatic developers to develop future tools.

## Materials and Methods

### Ethics Statement

The patients provided a written statement of informed consent. The research project was approved by the Ethics Committee of the Centre for Cardiovascular Surgery and Transplantation Brno.

### Mutations and Minigene Constructs

Two sequence variants that affect the first nucleotides in the *BTK* gene exons 15 and 18 were detected in patients with X-linked agammaglobulinaemia (see Table S1 in File S1). Other herein *in vitro* analyzed variants were sought in the Ensembl database, HGMD (The Human Gene Mutation Database; http://www.hgmd.cf.ac.uk) and RAPID (Resource of Asian Primary Immunodeficiency Diseases; http://rapid.rcai.riken.jp/RAPID). PCR products encompassing the mutated exons and at least 150 bp of the flanking intronic sequence or the 3′ UTR sequence were cleaved using appropriate restriction enzymes and cloned into the pET vector (MoBiTec, Göttingen, Germany). Sequence variants derived from literature were prepared by PCR-based artificial mutagenesis using specific primers harboring the desired mutation and the Pfx polymerase (Life Technologies, Carlsbad, CA). The quality of the inserted sequences was controlled by cycle sequencing on an ABI PRISM 3100-Avant Genetic Analyser (Life Technologies).

### *In silico* and other Sequence Analyses

The computational predictions were performed using the following web-based resources: the NNSplice [8], the MaxEnt [9], the PSSM [7], ESE-finder [14], RESCUE-ESE [12], Chasin ESE [13], Wang ESS [15], Chasin ESS [13] and BS and PPT predictions according to Kol et al. [10] and Schwartz et al. [11]. These tools, with the exception of NNSplice, were accessed using the Splicing Regulation Online Graphical Engine (Sroogle), and the score percentiles within constitutive exon datasets compiled and presented by this server were also used (http://sroogle.tau.ac.il/) [16]. All score and percentile differences between wild type (wt) and mutant variants were calculated as relative differences, e.g. as follows: (wt percentile – mutant percentile)/wt percentile.

In theory, changes of SRE that may overlap the E^+1^ position could play an important role in affecting the splicing fate of the exon in which they are located (for further explanation see discussion). However, considering that SRE predictions are rather complex and not all predicted changes would clearly affect splicing, we did not consider all SRE predicted changes (see discussion for more details). In fact, we considered predictions as positive only when the gains of exon splicing silencers (ESS) or losses of exon splicing enhancers (ESE) outnumbered the opposite changes of the respective elements. In addition, we performed SRE predictions twice: in one case we kept the borders of exons and introns when inserted in the Sroogle engine; in the other case we allowed the prediction tool to consider the whole sequence as an exonic one. The reason for this approach was that exonic elements are better explored than intronic ones, and binding of RNA binding proteins to these elements may occur before the exact definition of the intron/exon borders.

Since we used the PPS of 3′ss as a marked attribute of AG-dependence, we feel it necessary to illustrate the rules we used to define it. As the minimal binding sequence of each U2AF65-domain comprises of 4 nts [17], [18], [19], we considered this length as minimal to define PPS. Of these, we counted the longest uninterrupted stretch of Py that has at least one Py located between the third and the twentieth nucleotide from 3′ss. This choice of length was made because according to ours and previous results we do not expect farther located PY stretches to be involved in splice site definition. Therefore, this kind of localization definition was decided so that short Py stretches farther from the splice site were not counted, but at the same time did not exclude very long stretches. Note that according to herein mentioned rules one PPS (of sequence designated as “Fu11”, gene *CAPN3*, exon 17; see Table S1 in File S1) was counted differently in this work with respect to [3].

### Cell Culture and Transfection Procedures

HeLa cell line was obtained from the ATCC repository by the E. Baralle’s laboratory (LGC Standards S.r.l., Milan, Italy). U-937 cell line was purchased from DSMZ (Leibniz Institute DSMZ German Collection of Microorganisms and Cell Cultures, Braunschweig, Germany). HeLa and U-937 cell lines were maintained in RPMI 1640 medium (Sigma-Aldrich, Prague, Czech Republic) supplemented with 10% fetal calf serum (Zoo Servis, Huntirov, Czech Republic), 2 mM L-glutamine, 100 U/ml penicillin and 0.1 mg/ml streptomycin (Sigma-Aldrich). The HeLa cells (1×10^5^ cells per transfection experiment) were seeded into a 24-well plate one day prior to transfection. U-937 cells (3×10^5^ per transfection experiment) were seeded into a 24-well plate immediately prior to transfection. Both cell lines were transfected using 1.2 µl transfection reagent (XtremeGene 9 for HeLa cells and XtremeGene HP for U-937 cells; Roche Applied Science, Prague, Czech Republic) and 400 ng plasmid DNA per transfection experiment. The RNA was extracted 24 hours posttransfection.

### RNA Extraction

Total RNA from HeLa and U-937 cells was purified using an RNeasy Plus mini kit (Qiagen, Hilden, Germany). The quality and quantity of the RNA was determined by spectrophotometry (Thermo Scientific, Wilmington, DE) and its integrity was checked on a 1.5% agarose gel. Total RNA from peripheral blood stabilized in RNA*later* solution (Life Technologies) was extracted using a RiboPure-Blood Kit (Life Technologies) according to the manufacturer’s instructions.

### RT-PCR

RNA (10% of the total yield) extracted from the transfected cells was subjected to reverse transcription using random hexamers and a Transcriptor First Strand cDNA Synthesis Kit (Roche Applied Science, Prague, Czech Republic). The synthesized cDNA (5% of the total sample) was used as a template for PCR using recombinant Taq polymerase (Thermo Scientific, Erembodegem, Belgium) and the following pET vector specific primers, forward: 5′-CAGCACCTTTGTGGTTCTCA-3′, reverse: 5′-AGTGCCAAGGTCTGAAGGTC-3′. RT-PCR on RNA extracted from patients’ whole blood was performed using *BTK* gene specific primers (available upon request) and SuperScript One-Step RT-PCR with Platinum Taq (Life Technologies, Carlsbad, CA). The resulting fragments were visualized on 2% agarose gels. For quantification, the amplicons were resolved on a 4% denaturing polyacrylamide gel containing 7 M urea in 0.5 times Tris-acetate buffer and GeneTools software (Syngene, Cambridge, UK) was employed.

### Statistics

A median estimate supplied with a min-max range was used as robust summary statistics. The statistical significance of the differences between the computationally predicted scores for AG-dependent and AG-independent 3′ss was evaluated using the nonparametric Mann-Whitney test. The correlation between exon skipping and the predicted scores was assessed using Spearman’s rank correlation coefficient. The comparison of mutation frequency to T and other nucleotides, and of prediction of SRE changes in the AG-dependent and AG-independent 3′ss were both accomplished using Fisher’s exact test. The specificity and sensitivity estimates for the selected prediction tools were supplied with 95% confidence limits, calculated using Vassar College’s online Sensitivity/Specificity Calculator (http://faculty.vassar.edu/lowry/clin1.html). Statistical analysis was performed using STATISTICA software (version 10, StatSoft, Tulsa, OK). A value a = 0.05 was used as limit of statistical significance in all performed analyses.

## Results

**Figure 1:**
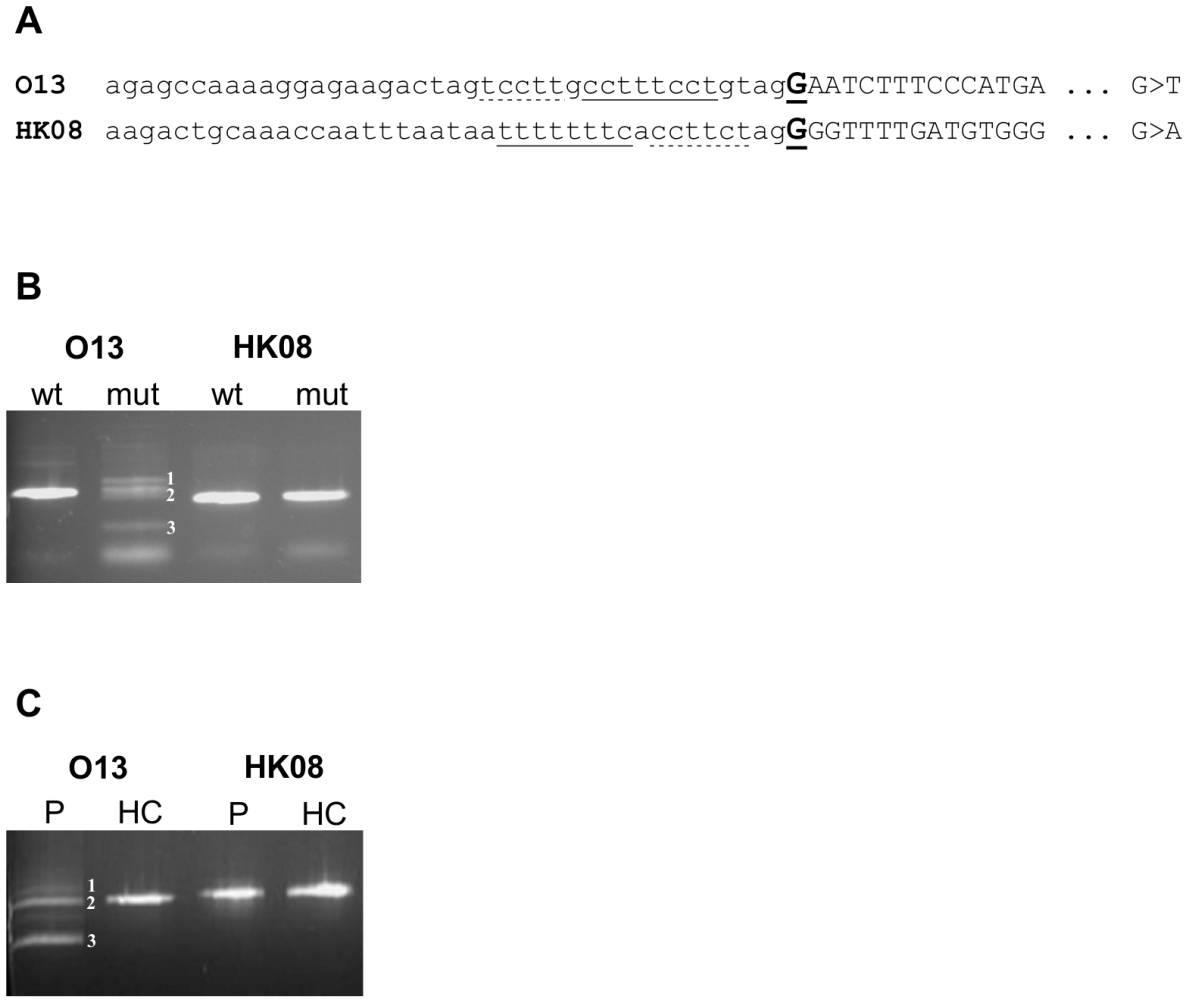
Analysis of the two E^+1^ mutations of the *BTK* gene, O13 (c.1482G>T) and HK08 (c.1883G>A). (A) Schematic sequences of mutated acceptor splice sites. Introns are shown in lower-case, and exons are shown in capital letters. Mutated nucleotides are bold and underlined. The PPS are singly underlined and other polypyrimidine stretches are dashed underlined. (B) RT-PCR of minigenes transfected into HeLa cells. cDNA bands originating from O13 mutated minigene are numbered as follows: 1) cryptic 3′ss utilization 81 nt upstream of the authentic splice site (the aberrant exon starts at c.1350-81G), 2) normally spliced RNA, 3) skipping of mutated exon. (C) RT-PCR from RNA extracted from patients’ blood. P = patient’s sample, HC = healthy control sample. The O13 cDNA bands are numbered as in (B).

Initially, we detected two variants that affected the first nucleotide of exons 15 and 18 in the *BTK* gene in our patients suffering from X-linked agammaglobulinaemia (Figure 1A, Table S1 in File S1). Using *in vitro* analyses, both splicing minigene assay (Figure 1B) and RT-PCR assay on RNA extracted from patients’ whole blood (Figure 1C) demonstrated that one of these variants (mutation c.1350G>T in exon 15, designated as “O13”) clearly affected splicing, whereas the other variant (mutation c.1751G>A at the beginning of exon 18, designated as “HK08”) did not. According to the results of Fu et al. [3], the 8 nucleotides long PPS of the mutated splice sites were just below the length range to score as AG-independent sites (that were shown to have PPS from 9 to 16 pyrimidines long). Even the results of the computer predictions were not very decisive (shown in the Tables S2 and S3 in File S1). In fact, all the predicted scores evaluating the overall 3′ss quality decreased when the E^+1^ mutations were introduced, with a greater decline observed for the O13 variant. By contrast, the PPT predictions did not conform to these data and depicted the O13 PPT to be of the same or even higher strength in comparison with the PPT of the HK08 sequence.

**Figure 2:**
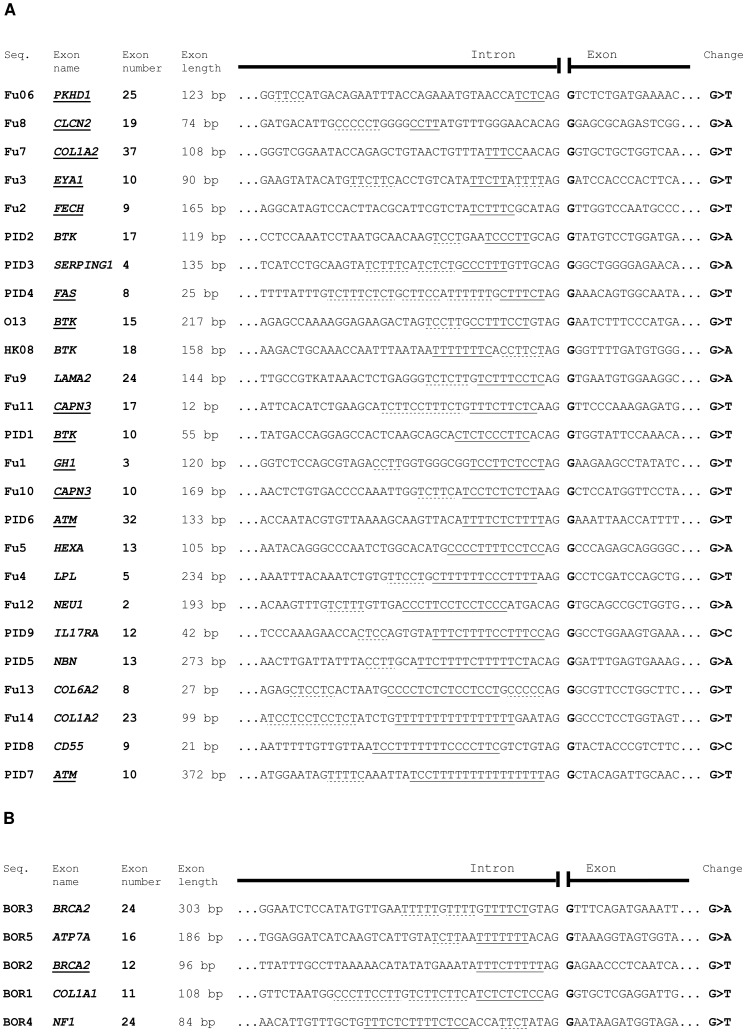
Analyzed sequences. The sequences are ordered according to the length of their longest PPS. Exons whose splicing was shown to depend on intact E^+1^ position are underlined. The PPS are singly underlined and other polypyrimidine stretches are dashed underlined. Sites of mutations are showed in bold. Seq. = sequence. (A) Sequences of the test set. (B) Sequences of the borderline set.

Because of this difference, we decided to evaluate the overall reliability of splicing prediction tools for E^+1^ mutations, using our mutations and a series of database-derived and previously analyzed E^+1^ variants [3]. To minimize interfering factors, we decided to preserve the design of the experiments made by Fu et al. [3] and to include only G^+1^ substitutions into our analyses. Using these criteria, we initially compiled three sets of mutations: the first set (named “test set”) of 25 sequence variants was used for primary analysis of *in silico* tools performance; the second set (named “Fu-mut set”) comprised of 30 non-natural sequence variants in which PPT was artificially mutated in [3]; and the third set (named “borderline set”) consisted of 5 natural database-derived sequence variants selected in such a manner that their PPS were of borderline quality (with regards to length and/or distance from 3′ss) to score as either AG-dependent or AG-independent (Figure 2 and S1 and Table S1 in File S1). These two latter sets were used to evaluate our results driven from primary analysis.

More specifically, the “test set” of mutations was obtained by combining all the 14 naturally occurring sequence variants analyzed by Fu et al. [3], 2 *BTK* mutations described above and 9 other variants derived from mutation databases. These 9 additional mutations were selected in the genes responsible for the development of primary immunodeficiencies (PID), 4 of them being presumably splicing-affecting and 5 others non-affecting, based on the length of their PPS (Table S1 in File S1).

**Figure 3:**
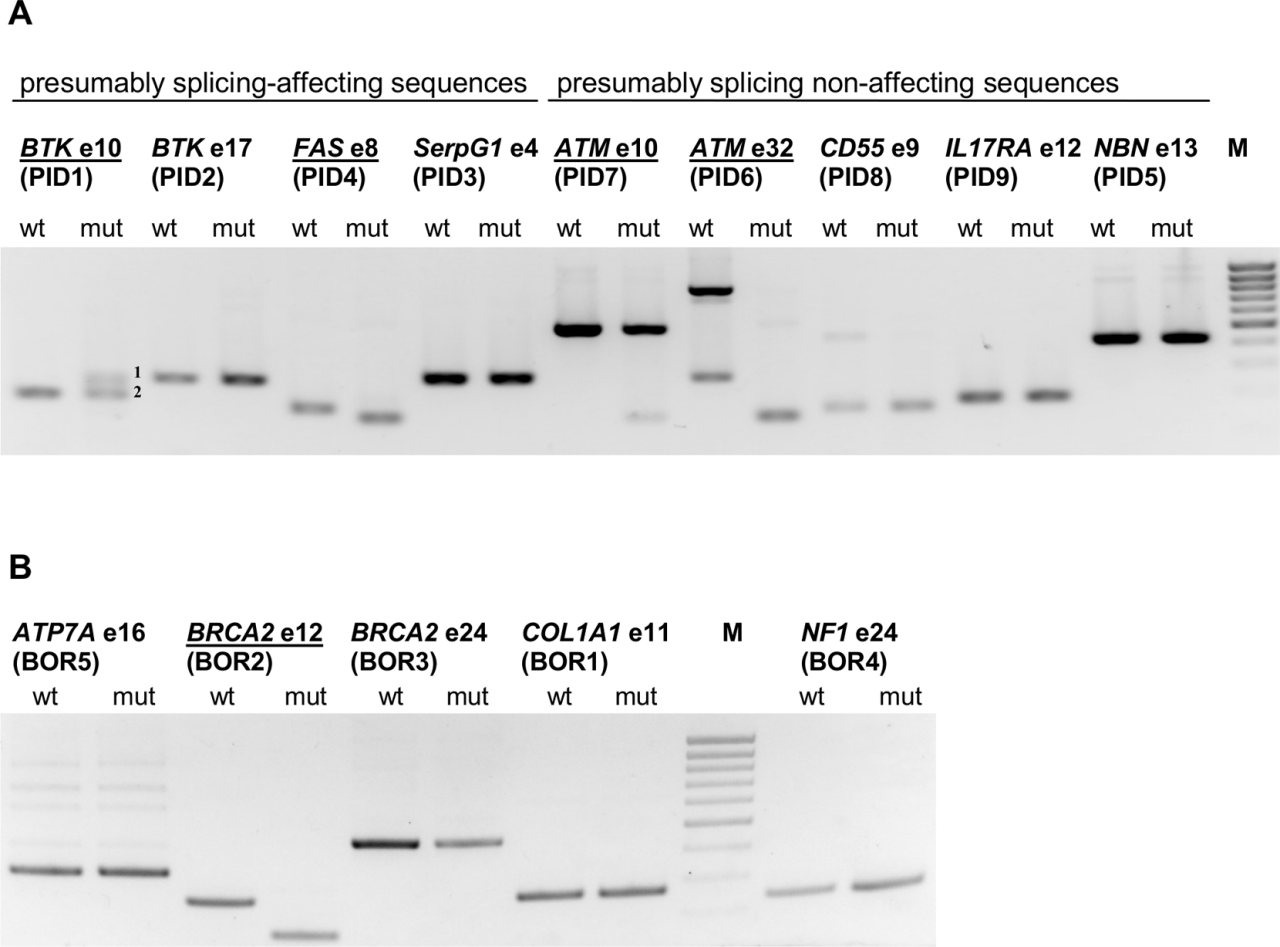
Results of the splicing minigene analyses. RT-PCR analysis of the literature-derived E^+1^ variations. The splicing affecting sequences are underlined. (A) The test set sequences. cDNA bands originating from *BTK* exon 10 mutated minigene are numbered as follows: 1) cryptic 3′ss utilization 31 nt upstream of the authentic splice site (the aberrant exon starts at c.840-31G), 2) normally spliced RNA. (B) The borderline set sequences.

All the novel variants were then analyzed using splicing minigene assay the same way as described for the *BTK* mutations. As shown in Fig. 3A, not all the supposedly splicing-affecting variants were capable of affecting the splicing process and vice versa. In particular, in accordance with our expectations *BTK* exon 10 (designated as PID1) and *FAS* exon 8 (PID4) variants did affect splicing whilst *CD55* exon 9 (PID8), *IL17RA* exon 12 (PID9) and *NBN* exon 13 (PID5) did not. On the other hand, *BTK* exon 17 (PID2) together with *SerpinG1* exon 4 (PID3) and *ATM* exon 10 (PID7) together with *ATM* exon 32 (PID6) did not conform with our assumptions, the latter ones showing that they affect splicing. In total, therefore, 13 sequences of the test set were shown to affect the splicing process in a minigene context, whilst the remaining 12 did not affect this process (see Table S1 in File S1 for details).

**Figure 4:**
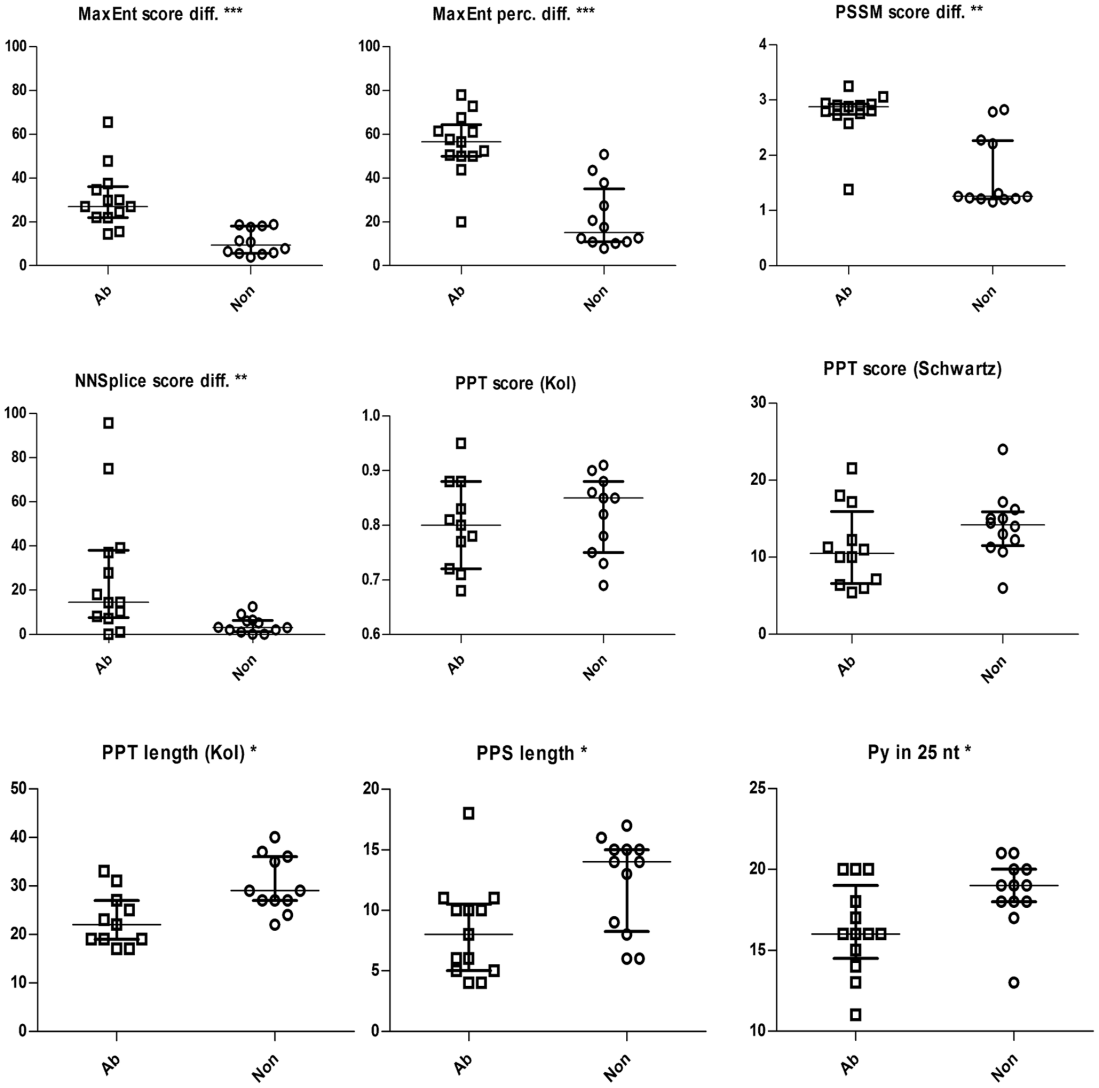
Selected results of *in silico* predictions and other parameters of AG-dependent and AG-independent splice sites. The boxplots show the values, median and interquartile range of the values predicted for the test set sequences. The values of scores are shown in the original units of the tools, differences are counted as ratios of the absolute score (or percentile) difference to the wild type score (or percentile). Other values are simply numbers of nucleotides. Asterisks indicate statistically significant differences between the two sets of values (*at p<0.05; **at p<0.01; ***at p<0.001). Abbreviations: Ab = group of sequence variants that lead to aberrant splicing; diff. = difference; Non = group of sequence variants that do not disrupt the process of splicing; nt = nucleotide(s); perc. = percentile; Py in 25 nt = number of pyrimidines in 25 nucleotides upstream of acceptor splice site.

In the predicted scores, percentiles and their differences between the wt and mutant sequences of the test set, we then searched for a distinction between the sequences vulnerable to the G^+1^ mutations and those that were not. The results of the predictions are summarized in Figure 4 and Tables 1 and 2 and depicted in detail in Tables S2, S3 and S4 in File S1). Our results demonstrated that the maximal discriminative power (the maximal statistical significance estimated using the Mann-Whitney test) was achieved using the MaxEnt tool, followed by the PSSM instrument. More specifically, the MaxEnt tool provided a good resolution of intron AG-dependence using mutant sequence scores and percentiles and, in particular, differences between wt and mutant sequence scores and percentiles. Note that all the predicted percentiles are derived from the Sroogle engine and pertain to the ranking of the predicted values in the dataset of more than fifty thousand constitutive exons [16]. The PSSM showed very similar outcomes, although with lower statistical significance than the MaxEnt tool. Even the NNSplice predictions discriminated between the splicing-affecting and non-affecting G^+1^ mutations using the predicted mutant sequence score and the score difference (Table 1).

**Table 1:** Predicted value ranges in the test set of G^+1^ mutations obtained from the instruments evaluating the overall strength of the 3′splice site.

	Splicing-affecting mutations (N = 13)			Non-affecting mutations (N = 12)			M-W test
	median	min	max	median	min	max	*P*
**NNSplice** wt score	0.96	0.08	1.00	0.98	0.92	1.00	0.073
**NNSplice** mut score	0.82	0.01	1.00	0.94	0.83	0.99	**0.011**
**NNSplice** score diff.	0.15	0.00	0.96	0.03	0.00	0.13	**0.005**
**MaxEnt** wt score	9.11	1.63	12.07	10.70	6.39	12.78	0.174
**MaxEnt** wt perc.	0.57	0.02	0.95	0.82	0.17	0.98	0.183
**MaxEnt** mut score	6.95	0.56	10.32	9.86	6.14	11.32	**0.003**
**MaxEnt** mut perc.	0.22	0.01	0.76	0.69	0.14	0.88	**0.003**
**MaxEnt** score diff.	0.27	0.14	0.66	0.09	0.04	0.19	**<0.001**
**MaxEnt** perc. diff.	0.57	0.20	0.78	0.16	0.08	0.51	**<0.001**
**PSSM** wt score	85.99	76.61	96.07	89.42	83.13	95.07	0.061
**PSSM** wt perc.	0.52	0.02	1.00	0.84	0.24	1.00	0.061
**PSSM** mut score	83.50	74.12	93.59	87.91	82.04	93.97	**0.024**
**PSSM** mut perc.	0.28	0.01	0.99	0.70	0.17	0.99	**0.026**
**PSSM** score diff.	0.03	0.01	0.03	0.01	0.01	0.03	**0.002**
**PSSM** perc. diff.	0.45	0.01	0.58	0.15	0.01	0.34	**0.003**

Computational predictions for experimentally confirmed test set of splicing-affecting and non-affecting G^+1^ mutations were subjected to statistical analysis using the Mann-Whitney test (see Table S1 File S1 for details). Significant differences (p<0.05) are marked in bold. Diff. = difference, M-W test = Mann-Whitney test, perc. = percentile.

**Table 2:** Comparison of value ranges describing particular intronic parameters in the test set of G^+1^ mutations.

	Splicing-affecting mutations(N = 13)			Non-affecting mutations(N = 12)			M-W test
	median	min	max	median	min	max	*P*
**PPS** length	8	4	18	14	6	17	**0.026**
**Py** in 25 bp	16	11	20	19	13	21	**0.034**
**Py** in 50 bp	29	21	37	34	26	38	0.053
**BS** score (Kol)	2.95	2.50	3.55	3.05	2.60	3.85	0.431
**BS** perc. (Kol)	0.25	0.04	0.84	0.36	0.07	0.98	0.431
**BS** to 3′ss dist. (Kol)	24	20	34	34	25	41	**0.009**
**BS** to PPT dist. (Kol)	0	0	6	0	0	5	0.844
**BS** score (Schwartz)	3	2	4	2.5	2	5	0.847
**BS** perc. (Schwartz)	0.05	0.00	0.32	0.19	0.00	0.32	0.847
**BS** to 3′ss dist. (Schwartz)	45	8	107	28.5	19	95	0.512
**BS** to PPT dist. (Schwartz)	24	2	88	14	−5	72	0.335
**PPT** length (Kol)	22	17	33	29	22	40	**0.010**
**PPT** to 3′ss dist. (Kol)	2	1	5	2	1	6	0.844
**PPT** score (Kol)	0.8	0.68	0.95	0.85	0.69	0.91	0.491
**PPT** perc. (Kol)	0.56	0.10	0.98	0.69	0.13	0.90	0.431
**PPT** length (Schwartz)	12.5	6	39	15.5	6	35	0.149
**PPT** to 3′ss dist. (Schwartz)	3.5	2	25	4	2	7	0.729
**PPT** score (Schwartz)	10.50	5.44	21.56	14.22	6.00	24.03	0.126
**PPT** perc. (Schwartz)	0.57	0.07	0.98	0.82	0.11	0.99	0.119

The BS and PPT parameters were predicted using prediction programs provided by Kol et al. [10] or Schwartz et al. [11] using the Sroogle engine. Computational predictions and other sequence parameters for an experimentally confirmed test set of splicing-affecting and non-affecting G^+1^ mutations were subjected to statistical analysis using the Mann-Whitney test (see Table S1 File S1 for details). Significant differences (p<0.05) are marked in bold. dist. = distance, BS = branch point site, M-W test = Mann-Whitney test, perc. = percentile, PPS = the longest uninterrupted polypyrimidine stretch Py = number of pyrimidines (in 25 or 50 nt upstream from 3′ss).

Finally, a somewhat lower discriminative power than that of the tools predicting overall strength of 3′ss was observed for the distance between the BS predicted according to Kol et al. and the acceptor site and for the PPT length predicted according to Kol et al. [10]. On the other hand, other parameters predicted for the BS did not exhibit statistically significant differences, and a number of the PPT predicted parameters (e.g. the scores and percentiles derived from both matrices) failed to discriminate between the two types of introns (Table 2).

In parallel to these considerations, in the test set of mutations we also assessed the distribution of additional parameters that were not predicted computationally (see Table 2 for full details). In particular, the observation of statistically significant differences in the length of the PPS and the number of pyrimidines in the 25 nt sequence upstream of the acceptor site between the splicing-affecting and non-affecting G^+1^ mutations was in agreement with the results reported by Fu et al. [3] (Table 2). In accordance with the same report, we did not observe a correlation between these parameters or the computationally predicted 3′ss parameters and the level of exon skipping in the set of 13 splicing-affecting G^+1^ mutations, with one single exception: the distance of BS from the 3′ss predicted according to Schwartz was correlated with the mutant sequence exon skipping, reaching a value of R = −0.59 (p = 0.04).

With regards to other factors that can also influence exon definition such as 5′ splice site (5′ss) strength and exon length we did not observe any difference in these parameters between the splicing-affecting and non-affecting mutations, even after combining the 5′ss score with the predicted 3′ss strength (data not shown).

However, a striking difference was evident when comparing the identity of mutant nucleotides: the majority of mutations to T led to aberrant splicing, whereas the majority of mutations to A did not result in any change (see Table S1 in File S1). When we tested this hypothesis using Fisher’s exact test, comparing the number of T versus non-T mutations in both groups of splicing events (affected and not affected) we reached significance at p = 0.0002. These data conform well to the evolutionary conservation of the exon first nucleotide. Analysis of the herein examined sequences of the test set and the borderline set (30 sequences in total) in 36 eutherian mammal species (using Ensembl database) showed strong G^+1^ conservation, and only changes to A were detected in the E^+1^ positions of some species. Moreover, a change to T was found only twice in total but in these cases the sequence was no more depicted as exonic (data not shown).

Another factor that impacts the definition of an exon is the presence of SREs. These elements have been shown to be more active in proximity to splice sites [20] and therefore their overlap with the E^+1^ position cannot be ruled out. In keeping with this view, a SRE spanning the 3′ss position has been already detected in the *SMN1* and *SMN2* genes [21]. In order to evaluate the possibility that some G^+1^ mutations affect splicing through changes in SRE elements rather than affecting U2AF35 binding to the splice sites we analyzed SRE predictions provided by Sroogle engine (see section material and methods for details) [16]. Using the approach that preserves the definition of exonic and intronic sequences prior to analysis, we observed four positive predictions among splicing-affecting mutations while there was only one such case among the non-affecting variants. Furthermore, when we indicated the whole sequence as exonic we detected more predictions of SRE changes among splicing-affecting mutations (eleven vs. two among the non-affecting variants), reaching statistical significance at p<0.01 (Fisher’s exact test; Table S5 in File S1 and Table S6). In conclusion, it appears that the disruption of SRE elements may, in parallel to intronic AG-dependence constitute another mechanism underlying splicing affection upon E^+1^ mutation (see discussion for further details).

Expectedly, some overlap was observed between the resulting value ranges for the splicing-affecting and non-affecting mutations even in the parameters showing statistically significant differences. Nevertheless, we tried to define cut-off values that could assort the results of our predictions into two groups according to the splicing affection. To establish such cut-off values, we firstly searched for an approximate cut-off value that could discriminate these two types of sequences in the test set with minimal overlap. For integral parameters we employed exactly that value as a discriminative cut-off. For other parameters, we found the two closest non overlapping values from both splicing-affecting and non-affecting group and counted the final cut-off value as their arithmetic mean. The predicted scores and percentiles below these cut-off values and the differences between the predicted values for wild type and mutant sequences above the cut-off values are supposed to pertain to variants prone to affect splicing. Table 3 shows the selected cut-off values for the following variables: longest PPS, the number of pyrimidines in 25 nt upstream of the acceptor site, and the seven best discriminating *in silico* predicted parameters. In addition, for these same sequence parameters, Figure S2 shows the discriminative power of various potential cut-off values to discern between splicing affecting and non-affecting mutations in the test set sequences.

**Table 3:** Proposed cut-off values for the *in silico* tools that discriminate AG-dependent 3′ss from AG-independent 3′ss.

	Cut-off^a^	Sensitivity^b^		Specificity^b^		Sensitivity^c^		Specificity^c^	
		(N = 30)	95% confidence interval	(N = 30)	95% confidence interval	(N = 25)	95% confidence interval	(N = 25)	95% confidence interval
The longest **PPS**	**12**	65%	0.41–0.84	40%	0.14–0.73	92%	0.62–1.00	67%	0.35–0.89
**Number of Py** in 25 nt	**18**	70%	0.46–0.87	70%	0.35–0.92	69%	0.39–0.90	83%	0.51–0.97
**NNSplice** score difference	**6.83%**	50%	0.28–0.72	50%	0.20–0.80	85%	0.54–0.97	83%	0.51–0.97
**MaxEnt** mut score	**8.07**	95%	0.73–1.00	70%	0.35–0.92	85%	0.54–0.97	83%	0.51–0.97
**MaxEnt** mut perc.	**0.38**	95%	0.73–1.00	70%	0.35–0.92	85%	0.54–0.97	83%	0.51–0.97
**MaxEnt** score difference	**20.00%**	90%	0.67–0.98	70%	0.35–0.92	85%	0.54–0.97	100%	0.70–1.00
**MaxEnt** perc. difference	**43.76%**	84%	0.60–0.96	60%	0.27–0.86	92%	0.62–1.00	92%	0.60–1.00
**PSSM** score difference	**2.43%**	75%	0.51–0.90	30%	0.08–0.65	92%	0.62–1.00	83%	0.51–0.97
**PSSM** perc. difference	**30.43%**	68%	0.43–0.86	40%	0.14–0.73	77%	0.46–0.94	92%	0.60–1.00

a The cut-off values were proposed according to the predicted values obtained using the test set of naturally occurring G^+1^ mutations (as used in Tables 1 and 2). ^b^The sensitivity and specificity calculated using Fu-mut set of G^+1^ mutations, containing artificially mutated PPTs. ^c^The sensitivity and specificity calculated using the original test set of G^+1^ mutations. The predicted scores and percentiles below the cut-off values and the differences between the predicted values for wild type and mutant sequences above the cut-off values are supposed to pertain to variants prone to affect splicing.

Next, we tested these cut-off values for their ability to predict the mutation-induced splicing outcome. For this purpose, we examined the two evaluation sets described above: the Fu-mut set and the borderline set. Using 30 sequences of the Fu-mut set, the MaxEnt program produced the best results with up to 95% sensitivity and up to 70% specificity (Table 3). Using the borderline set in which *in vitro* analyses depicted just one of the five mutations as splicing-affecting, most of the cut-off values differentiated well between the two types of mutations.

Finally, seeking for a reasonable solution that we could offer to other scientists and diagnosticians working with E^+1^ mutations, we searched for a combination of predicted parameters that could maximize their discriminative power. For this purpose, in order to start from more data, we joined the predictions for the test set and the borderline set together. As a result, the best performing combinations of predicted values were: number of pyrimidines in 25 nucleotides upstream from 3′ss together with i) MaxEnt score difference and MaxEnt percentile difference; ii) MaxEnt score difference and PSSM score difference; iii) MaxEnt percentile difference and PSSM score difference. Using these combinations with the integrated set of sequences we gained up to 93% sensitivity and 94% specificity and we reached up to 95% sensitivity and 90% specificity when analyzing the highly artificial Fu-mut set (Table 4).

**Table 4:** Results of combined predictions in discrimination of G^+1^-dependent 3′ss from G^+1^-independent 3′ss.

Combined toolspredictions	Number of values	Sensitivity^b^		Specificity^b^		Sensitivity^c^		Specificity^c^	
	exceeding cut-off^a^	(N = 30)	95% confidence interval	(N = 30)	95% confidence interval	(N = 30)	95% confidence interval	(N = 30)	95% confidence interval
**A) Py25, ME s.d., ME p.d.**	**2**	95%	0.72–1.00	90%	0.54–0.99	93%	0.64–1.00	94%	0.68–1.00
**B) Py25, ME s.d., PSSM s.d.**	**2**	90%	0.67–0.98	70%	0.35–0.92	93%	0.64–1.00	94%	0.68–1.00
**C) Py25, ME p.d., PSSM s.d.**	**2**	89%	0.65–0.98	70%	0.35–0.92	93%	0.64–1.00	94%	0.68–1.00

a Each combined prediction was considered positive if two or more of the three predicted values exceeded the herein proposed cut-off values of the individual tools. ^b^The sensitivity and specificity was calculated using Fu-mut set of G^+1^ mutations, containing artificially mutated PPTs. ^c^The sensitivity and specificity calculated using combined sets: test set and borderline set of G^+1^ mutations. Py25 = number of pyrimidines in the 25 nucleotides upstream from splice site; ME s.d. = difference between wild type and mutant sequence scores predicted by MaxEnt program; ME p.d. = difference between wild type and mutant sequence percentiles predicted by MaxEnt program; PSSM s.d.: accordingly.

In order to provide further evaluation of these combined predictions on naturally occurring sequence variants, we derived 9 other G^+1^ mutations with a known effect on splicing from literature (Figure S3). The approach of combined predictions led to correct prediction of splicing affection in 7, 8 or all 9 cases according to the particular combination of tools used (Table S7 in File S1). We provide an excel table with ready-set formulae to facilitate making conclusions of the individual and the combinatorial predictions (Table S8).

## Discussion

In this study, we have examined *in vitro* the effect of 16 naturally occurring G^+1^ sequence variants on splicing and investigated the efficiency of *in silico* instruments to predict their ability to disrupt the pre-mRNA splicing process. In accordance with previous findings [3], we showed that the quality of the PPT is important but not the only factor determining the splicing dependency on an intact E^+1^ position. In addition, we also showed that the specific PPT prediction tools were not as efficient at predicting splicing outcomes as the instruments that evaluated the overall strength of the acceptor site.

In total, we have newly detected splicing affection in six G^+1^ variants. Remarkably, two of these variants, namely BOR2 (c.6842G>T; p.Gly2281Val) in the exon 12 of *BRCA2* gene and PID7 (c.1236 G>T; p.Trp412Cys) in the exon 10 of *ATM* are recorded in the dbSNP database as polymorphisms with unknown frequency and clinical significance.

The quality of the PPT depends on its overall length, its distance from the 3′ss, the number of pyrimidines and the number of consecutive uridines [22], [23]. Notably, for the series of human 3′ss investigated by Fu et al. [3], the authors suggest that the length of the PPS represents the best discriminating feature between AG-dependent and AG-independent introns; namely, the PPS was shown to encompass between 4 and 10 nt in the AG-dependent introns and between 9 and 16 nt in the AG-independent introns in the sequences analyzed in their experiments. This implies that the sequences with PPS of 10 nt or shorter have higher propensity to be aberrantly spliced upon E^+1^ mutation and deserve more detailed *in vitro* analysis [3].

When we reanalyzed these results after adding these 16 newly analyzed sequences to the Fu’s original set, the PPS of AG-dependent introns measured between 4 and 11 nt (with one outlier 18 nt long discussed below), and the PPS of AG-independent introns measured between 6 and 17 nt. Despite the significant difference between median values (8 nt for AG-dependent and 14 nt for AG-independent introns), the PPS length showed marked overlap. Notably, the AG-independent exons with shorter PPS generally had more than one polypyrimidine stretch in the PPT, indicating that even a few shorter stretches can assure AG-independence.

Among the number of features that influence the exon definition, we would like to emphasize the importance of SREs. In this work, we also considered the predicted ESE disruption or ESS creation spanning the E^+1^ position, as the events potentially affecting splicing.

From a mechanistic point of view, the ESE disruption and ESS creation differ significantly from each other. A protein factor bound to a newly created ESS (e.g. a protein from hnRNP family) could form a steric hindrance for U2AF35 binding [24]. Or, possibly, such a protein could multimerize along the RNA and block the binding of other factors, e.g. U2AF65, SF1, etc. [25]. In fact, both these events were proposed in the splicing regulation of *SMN1* and *SMN2* exon 7, where an ESS presence across the 3′ss was shown [21]. Alternatively, ESS-bound protein could hamper the function of a downstream ESE [24]. All these events could clearly impair the process of 3′ss recognition and cause the splicing aberration.

However, the effects of disruption of an ESE spanning the 3′ss could be less clear, since the presence of ESE in this position may not seem logical at first glance. The reason is that most of the ESE-binding proteins were shown to facilitate 3′ss recognition through interaction with U2AF35 which can then bind to the 3′ss more easily (see [26] for review). However, it was shown that some ESE-binding SR-proteins can interact directly with the U2AF65 or with U2 snRNP [27], [28], and for this reason these factors could promote splicing even in the absence of U2AF35.

In this respect, p54 protein from an SR-family that interacts directly with the U2AF65 was suggested to be able to functionally replace U2AF35 in its bridging other SR-proteins with U2AF65 [27]. Might be that this or similar proteins could act in place of U2AF35 in the 3′ss recognition that is essential for AG-dependent introns. In concordance with this, not all the AG-dependent introns were shown to depend on U2AF35 binding [3], [29]. Therefore, disruption of binding site for such a protein could impair U2AF65 binding to the PPT, thus precluding correct splice site recognition.

The other possible function of an ESE spanning the 3′ss position might be the stabilization of U2 binding to the branch point site. It was demonstrated that the RS-domain of an SR-protein contacts the BS in the prespliceosome and that such a contact helps in promoting the prespliceosome assembly [28], [30]. In fact, such an event occurs later in the spliceosome assembly than the original recognition of 3′ss by the U2AF complex and is probably enabled by the lower association of U2AF with RNA in the later spliceosomal complexes [31]. Therefore, disruption of an ESE could harm the splicing independently on U2AF35 binding. In addition, at least in AG-independent introns, an ESE spanning the 3′ss could antagonize the function of proximal ESS, analogically to the situation in the *SMN1* exon 7 [21].

In conclusion, therefore, we must consider the possibility that every E^+1^ mutation can affect not only the definition of a splice site, but may also introduce relevant changes in SRE elements that could lead to a splicing aberration even in the AG-independent splice sites. Furthermore, many described SREs are purine-rich elements [32] which might provide an explanation as to why the G to A changes in the E^+1^ position tend to be more easily tolerated by the splicing machinery than G to T changes in our analysis. For these reasons, we propose calling the sequences in which E^+1^ mutation affected splicing as “E^+1^-dependent” and the other sequences as “E^+1^-independent” rather than AG-dependent or independent, unless AG-dependence is demonstrated.

In keeping with this conclusion, SRE disruption has already been proven in the case of the herein analyzed GH1 exon 3 mutation (Fu01, c.172 G>T) [33]. In addition, mutation c.517G>T in the *BRCA2* exon 7 (Eval1) lies in a region where a splicing enhancer was previously detected [34]. In fact, disruption of a functional splicing enhancer and/or creation of a splicing silencer could provide an explanation for the cases of splicing affection in the introns with a good quality PPS and strong splice sites, such as in the cases of *ATM* exon 10, *BRCA2* exon 12 and *CFTR* exon 4 mutations (designated as PID7, BOR2 and EVAL4, respectively). Accordingly, potentially harmful SRE changes were predicted in all these three cases. Furthermore, we detected significantly higher occurrence of SRE changes between E^+1^-dependent sequences than in the E^+1^-independent ones.

Nonetheless, the predictions of the SREs encompassing the E^+1^ position were only partly helpful in discerning the E^+1^-dependent from E^+1^-independent sequences. In fact, although we detected a difference between the number of positive predictions of supposedly relevant SRE changes between the two groups of E^+1^-dependent and E^+1^-independent sequences, we suppose that these predictions overestimated the number of functional SRE changes. This is quite a common occurrence and it has been described several times that SRE predictions suffered from a high number of false positive and false negative results [35], [36].

Most importantly, as splicing dependence on the intact E^+1^ position is difficult to predict from sequence inspection alone, we examined the ability of available software tools to predict exonic E^+1^ dependence. Interestingly, we obtained the most promising results using the tools that estimated the overall quality of the splice site (i.e., MaxEnt, PSSM and NNSplice). The best predictions were obtained with the MaxEnt program, which proved to be highly efficient in previous studies as well [37], [38]. These results are consistent with the MaxEnt program basing its predictions on calculations of the interdependence of non-adjacent positions, which may correspond well to the cooperation of several signal sequences during splice site recognition [9].

In contrast, the outcomes of PPT predictions were not optimal. In fact, the predicted value ranges between E^+1^-dependent and E^+1^-independent groups of sequences overlapped considerably. The BS predictions produced similar results, which may correspond to the looser connection between the quality of the BS and the AG-dependence of the 3′ss [22]. However, the low performance of the PPT predictors is more remarkable because this element is believed to control the intron AG-dependence and, as explained above, our results confirm it to be major determinant of E^+1^ dependence. The most likely explanation is that this tool does not take into account the cooperation of factors bound to PPT and to other elements such as the splice site, BS or SREs [26], [39], [40].

Finally, in order to facilitate the work of genetic diagnosticians who often struggle with unclassified sequence variants whose action on the splicing process may be unclear, we have used our results to define discriminating border values for the software tools that might help assess whether a variant will be likely to influence splicing or not. Other authors have proposed that a 10% difference between the wild type- and mutant-predicted scores is an acceptable cut-off value [41], [42]. However, this value is neither suitable for nor applicable to all of the instruments tested (e.g., PSSM, see Tables S2, S3 and S4 in File S1). Although other authors have proposed that cut-off values might be calculated to become gene specific [41], an alternative could be to obtain cut-off values that might be specific for a particular splicing signal and the position of the mutation.

In accordance to this view, for our sets of G^+1^ mutations, we have proposed specific cut-off values that will allow E^+1^-dependent from E^+1^-independent sequences to be distinguished with reasonably high sensitivity and specificity. An exception was the case of BOR4 mutation (*NF1* exon 24; see Table S2 in File S1) that was predicted as splicing-affecting by most of the tools, while it did not affect splicing in fact. A possible explanation for this failure could be a rather specific, 14-nt long PPS located quite far from the 3′ss that may lead the prediction tools to mistakenly consider this splice site as weak.

Nonetheless, owing to different underlying algorithms, individual prediction tools have distinct advantages and disadvantages. Therefore, from our analysis we have further extracted three combinations of prediction parameters that when taken together, perform even better than the individual cut-off values.

Furthermore, in addition to their good results when evaluated with the set of sequences with artificially mutated PPT, these combined predictions performed well even with the natural sequence variants. For example, in the set of 9 evaluation sequences, the length of the PPS itself would have lead to misprediction of E^+1^-dependence in 7 cases, and even the individual *in silico* tools predictions would have failed in 19% of the predictions (Table S7 in File S1). However, when we applied combined predictions the correct splicing outcome could be predicted in all cases.

We therefore suggest that the proposed individual MaxEnt and PSSM cut-off values and their combinations should be considered when evaluating the potential splicing effects of new G^+1^ mutations. To facilitate work using the cut-off limits, we now add a simple calculation table as a supplement to this article (Table S8).

In conclusion, therefore, we believe that this work could serve as a preliminary guide for estimating the impact of exon first nucleotide mutations on splicing.

## Supporting Information

Figure S1 **Complete list of the sequences used for evaluation of** ***in silico*** **prediction tools effectivity.** Exons which splicing was shown to depend on intact E^+1^ position are underlined. The PPS are simply underlined, other polypyrimidine stretches are dashed underlined. Sites of mutations are showed in bold. Note that this figure contains all the herein used sequences except from artificially mutated ones of the Fu-mut sets, i.e. the test set, borderline set and the evaluation set.
(TIFF)

Figure S2 **Discriminative power of various potential cut-off values.** The charts show percentage of correctly sorted test set sequences according to mutation induced splicing affection (success rate) plotted against various cut-off values of individual sequence parameters. The dashed line and the number beside show finally selected cut-off value (selected according to the rules explained in results part). Note that the predicted scores and percentiles below these cut-off values and the differences between the predicted values for wild type and mutant sequences above the cut-off values are supposed to pertain to variants prone to affect splicing. Diff. = difference, perc. = percentile, Py25 = number of pyrimidines in the 25 nucleotides upstream from splice site.
(TIF)

Figure S3 **Sequences of the evaluation set.** Exons which splicing was shown to depend on intact E^+1^ position are underlined. The PPS are simply underlined, other polypyrimidine stretches are dashed underlined. Sites of mutations are showed in bold.
(TIFF)

Table S6 **Individual SRE predictions.** The individual predictions for all the herein analyzed sequences obtained at Sroogle engine. The table is divided into two parts, one with borders of exons and introns being kept when inserted in the Sroogle engine; the other with the whole sequence considered as an exonic one. Mut = mutant, wt = wild type. Other abbreviations as well as the colors identifying SRE types (red for enhancers, green for silencers and gray for nonspecified regulators) were adopted from Sroogle [S4].
(XLSX)

Table S8 **Ready-set formulae to facilitate making conclusions of the individual and the combinatorial predictions.** Acc = acceptor splice site; Longest Py stretch = the longest uninterrupted polypyrimidine stretch; Py = number of pyrimidines (in 25 upstream from 3’ss).
(XLS)

File S1 **Includes Tables S1–S5 and S7. Table S1. List of analyzed E^+1^ mutations of the test set and the borderline set.** Exon skipping analyses were described in this work for mutations O13, HK08, all the PID and all the BOR sequences, the rest was adopted from Fu et al. [S1]. The sequences that presented aberrant splicing following mutation are highlighted in light orange. The exons highlighted in yellow were selected as presumably splicing-affecting (the length of their PPS being 10 nt at maximum, containing the uninterrupted T-stretch not longer than 3 nt) and those highlighted in green as presumably splicing non-affecting (the minimal length of their PPS being 11 nt, with T-stretches of 4 nt at minimum; see results section). The depicted protein change is a predicted change based solely on DNA-level knowledge. The number of pyrimidines upstream from the 3’ss was counted in 25- (50-) nt sequences. The values that do not fall (according to the mutation influence on splicing) into the range of herein proposed cut-off limits are marked in blue. BOR = sequences from the “borderline set” of mutations. Py = number of pyrimidines, PPS = the longest uninterrupted polypyrimidine stretch, RefSeq = reference sequence in the NCBI database, skip. = ratio of exon skipping in the minigene analyses of wild type (wt) or mutant (mut) sequences. * there are several discrepancies in the numbering of exons in the reference sequences compared to the numbering shown in Fu et al. (2011): *EYA1* exon 10 is depicted as number 12 in the reference sequence; *CAPN3* exons 10 and 17 are depicted as numbers 5 and 12 in the reference sequence, respectively. ** mutation c.1350G>T has not been reported yet. *** the mutation was derived from RAPID database where mutations of G to A, C and T were depicted in the same position. However, the change selected for this article, G>A, was later found to be mistakenly obtained from [S2], where a mutation at adjacent position was described. **Table S2. Predicted values for the E^+1^ mutations using instruments evaluating the overall strength of the 3’splice site.** The values that do not fall (according to their sequences influence on splicing) into the range of the herein proposed cut-off limits are marked in blue. The sequences that were shown to adopt aberrant splicing upon mutation are highlighted in light orange. Diff. = difference, perc. = percentile, seq. = sequence **Table S3. Predicted values for the PPT of the E^+1^ mutated sequences.** The sequences that were shown to adopt aberrant splicing upon mutation are highlighted in light orange. “–” indicates the cases where the computer tool gave no values. Perc. = percentile, dist. = distance **Table S4.** **Predicted values for the BS of the E^+1^ mutated sequences.** The sequences that were shown to adopt aberrant splicing upon mutation are highlighted in light orange. “–” indicates the cases where the computer tool gave no values. Perc. = percentile, dist. = distance **Table S5. Prediction of SRE changes (using Sroogle engine).** The sequences that were shown to adopt aberrant splicing upon mutation are highlighted in light orange. Positive predictions are marked in green. For an explanation, see material and methods. The statistical comparison of the SRE changes in splicing-affecting and non-affecting samples was counted only from the test-set of sequences (i.e. without the borderline set sequences). **Table S7. Combined predictions of splicing affection on nine evaluation sequences.** ^a^ Each combined prediction was considered as positive if two (or more) of the three predicted values exceeded the herein proposed cut-off values of the individual tools. The individual values that do not fall into the range of herein proposed cut-off limits are marked in blue. Predictions being in accordance with detected splicing affection are marked in green, the discrepancies are in orange. Py25 = number of pyrimidines in the 25 nucleotides upstream from splice site; ME s.d. = difference between wild type and mutant sequence scores predicted by MaxEnt program; ME p.d. = difference between wild type and mutant sequence percentiles predicted by MaxEnt program; PSSM s.d.: accordingly.
(DOC)
