# Supplementary figures and images for: Exon First Nucleotide Mutations in Splicing: Evaluation of In Silico Prediction Tools
Source: PLoS One. 2014 Feb 21;9(2):e89570. doi: 10.1371/journal.pone.0089570 (PMC3931810; doi:10.1371/journal.pone.0089570)

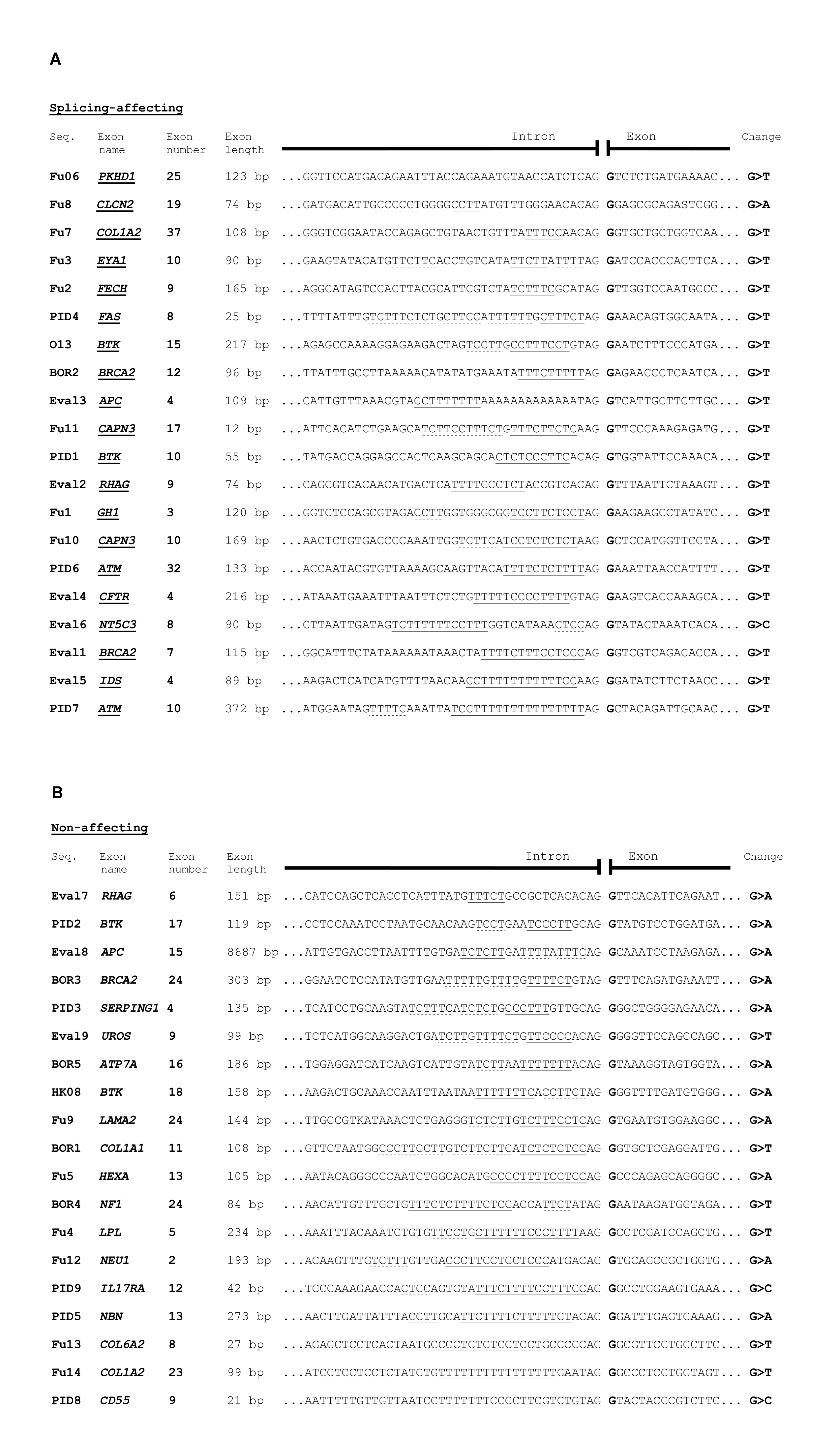

Supplement: Figure S1 — Complete list of the sequences used for evaluation of in silico prediction tools effectivity. Exons which splicing was shown to depend on intact E+1 position are underlined. The PPS are simply underlined, other polypyrimidine stretches are dashed underlined. Sites of mutations are showed in bold. Note that this figure contains all the herein used sequences except from artificially mutated ones of the Fu-mut sets, i.e. the test set, borderline set and the evaluation set. (TIFF) [file pone.0089570.s001.tiff]

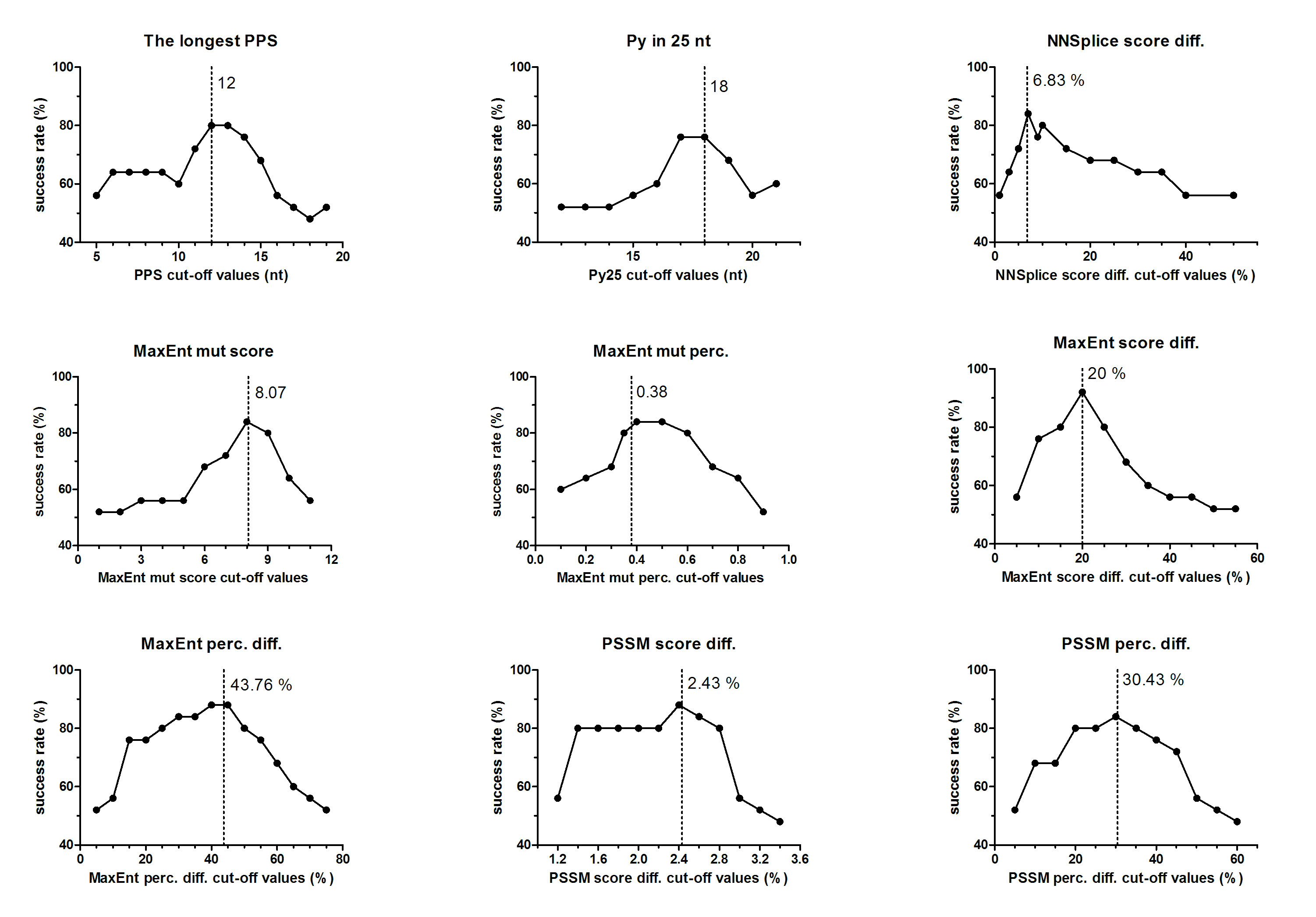

Supplement: Figure S2 — Discriminative power of various potential cut-off values. The charts show percentage of correctly sorted test set sequences according to mutation induced splicing affection (success rate) plotted against various cut-off values of individual sequence parameters. The dashed line and the number beside show finally selected cut-off value (selected according to the rules explained in results part). Note that the predicted scores and percentiles below these cut-off values and the differences between the predicted values for wild type and mutant sequences above the cut-off values are supposed to pertain to variants prone to affect splicing. Diff. = difference, perc. = percentile, Py25 = number of pyrimidines in the 25 nucleotides upstream from splice site. (TIF) [file pone.0089570.s002.tif]

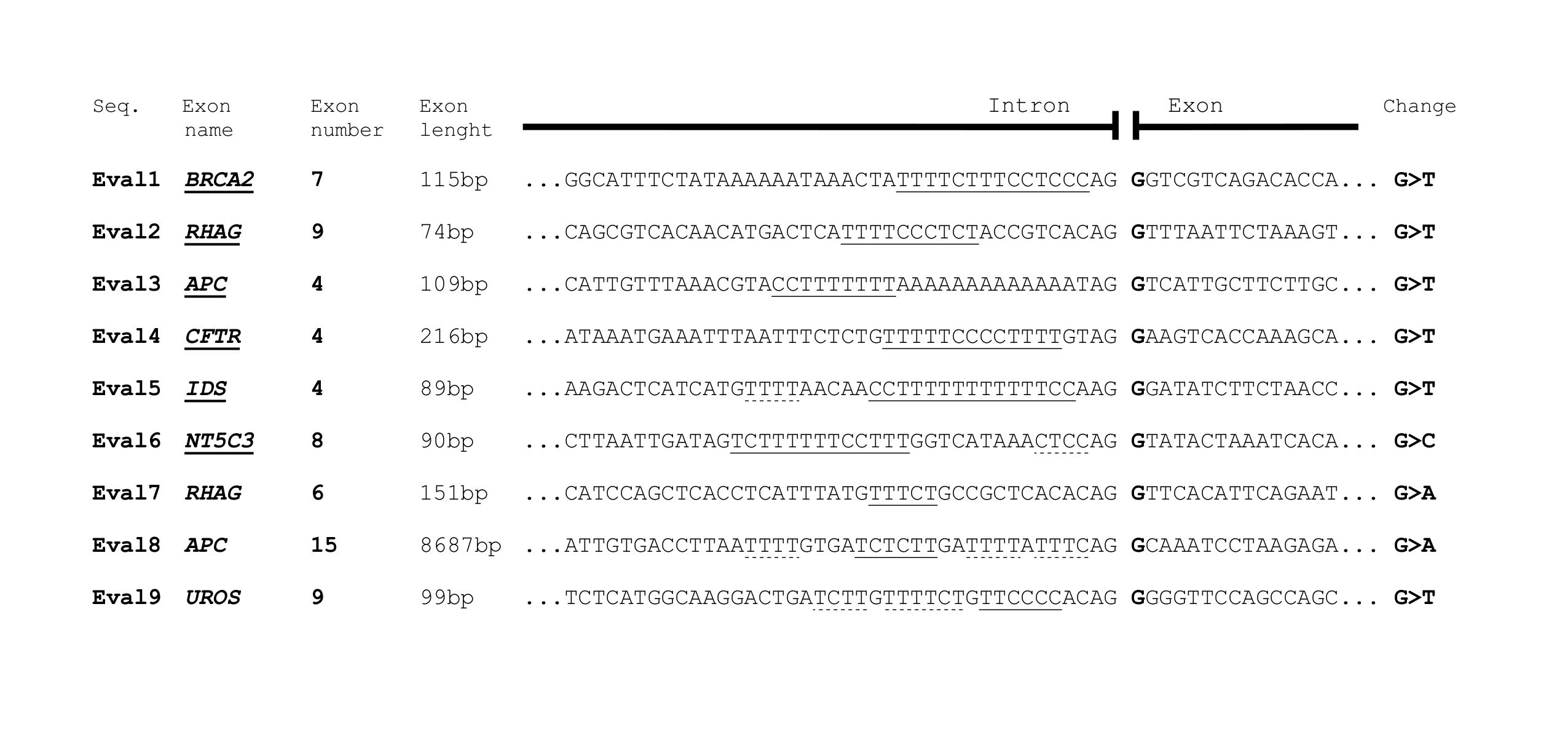

Supplement: Figure S3 — Sequences of the evaluation set. Exons which splicing was shown to depend on intact E+1 position are underlined. The PPS are simply underlined, other polypyrimidine stretches are dashed underlined. Sites of mutations are showed in bold. (TIFF) [file pone.0089570.s003.tiff]
