# Supplementary material for: Exon First Nucleotide Mutations in Splicing: Evaluation of In Silico Prediction Tools
Source: PLoS One. 2014 Feb 21;9(2):e89570. doi: 10.1371/journal.pone.0089570 (PMC3931810; doi:10.1371/journal.pone.0089570)
Supplement: File S1 — Includes Tables S1–S5 and S7. Table S1. List of analyzed E+1 mutations of the test set and the borderline set. Exon skipping analyses were described in this work for mutations O13, HK08, all the PID and all the BOR sequences, the rest was adopted from Fu et al. [S1]. The sequences that presented aberrant splicing following mutation are highlighted in light orange. The exons highlighted in yellow were selected as presumably splicing-affecting (the length of their PPS being 10 nt at maximum, containing the uninterrupted T-stretch not longer than 3 nt) and those highlighted in green as presumably splicing non-affecting (the minimal length of their PPS being 11 nt, with T-stretches of 4 nt at minimum; see results section). The depicted protein change is a predicted change based solely on DNA-level knowledge. The number of pyrimidines upstream from the 3’ss was counted in 25- (50-) nt sequences. The values that do not fall (according to the mutation influence on splicing) into the range of herein proposed cut-off limits are marked in blue. BOR = sequences from the “borderline set” of mutations. Py = number of pyrimidines, PPS = the longest uninterrupted polypyrimidine stretch, RefSeq = reference sequence in the NCBI database, skip. = ratio of exon skipping in the minigene analyses of wild type (wt) or mutant (mut) sequences. * there are several discrepancies in the numbering of exons in the reference sequences compared to the numbering shown in Fu et al. (2011): EYA1 exon 10 is depicted as number 12 in the reference sequence; CAPN3 exons 10 and 17 are depicted as numbers 5 and 12 in the reference sequence, respectively. ** mutation c.1350G>T has not been reported yet. *** the mutation was derived from RAPID database where mutations of G to A, C and T were depicted in the same position. However, the change selected for this article, G>A, was later found to be mistakenly obtained from [S2], where a mutation at adjacent position was described. Table S2. Predicted values for [file pone.0089570.s006.doc]

Title:

**Exon first nucleotide mutations in splicing: evaluation of *in silico* prediction tools**

Authors: Lucie Grodecká, Pavla Lockerová, Barbora Ravčuková, Emanuele Buratti, Francisco E. Baralle, Ladislav Dušek, Tomáš Freiberger

**Supplemental tables**

**Table S1. List of analyzed E^+1^ mutations of the test set and the borderline set.**

Exon skipping analyses were described in this work for mutations O13, HK08, all the PID and all the BOR sequences, the rest was adopted from Fu et al. [S1]. The sequences that presented aberrant splicing following mutation are highlighted in light orange. The exons highlighted in yellow were selected as presumably splicing-affecting (the length of their PPS being 10 nt at maximum, containing the uninterrupted T-stretch not longer than 3 nt) and those highlighted in green as presumably splicing non-affecting (the minimal length of their PPS being 11 nt, with T-stretches of 4 nt at minimum; see results section). The depicted protein change is a predicted change based solely on DNA-level knowledge. The number of pyrimidines upstream from the 3'ss was counted in 25- (50-) nt sequences. The values that do not fall (according to the mutation influence on splicing) into the range of herein proposed cut-off limits are marked in blue. BOR = sequences from the “borderline set” of mutations. Py = number of pyrimidines, PPS = the longest uninterrupted polypyrimidine stretch, RefSeq = reference sequence in the NCBI database, skip. = ratio of exon skipping in the minigene analyses of wild type (wt) or mutant (mut) sequences.

* there are several discrepancies in the numbering of exons in the reference sequences compared to the numbering shown in Fu et al. (2011): *EYA1* exon 10 is depicted as number 12 in the reference sequence; *CAPN3* exons 10 and 17 are depicted as numbers 5 and 12 in the reference sequence, respectively.

** mutation c.1350G>T has not been reported yet.

*** the mutation was derived from RAPID database where mutations of G to A, C and T were depicted in the same position. However, the change selected for this article, G>A, was later found to be mistakenly obtained from [S2], where a mutation at adjacent position was described.

**Table S2. Predicted values for the E^+1^ mutations using instruments evaluating the overall strength of the 3'splice site.**

The values that do not fall (according to their sequences influence on splicing) into the range of the herein proposed cut-off limits are marked in blue. The sequences that were shown to adopt aberrant splicing upon mutation are highlighted in light orange.

Diff. = difference, perc. = percentile, seq. = sequence

**Table S3. Predicted values for the PPT of the E^+1^ mutated sequences.**

The sequences that were shown to adopt aberrant splicing upon mutation are highlighted in light orange.

“-“ indicates the cases where the computer tool gave no values. Perc. = percentile, dist. = distance

**Table S4.** **Predicted values for the BS of the E^+1^ mutated sequences.**

The sequences that were shown to adopt aberrant splicing upon mutation are highlighted in light orange.

“-“ indicates the cases where the computer tool gave no values. Perc. = percentile, dist. = distance

**Table S5. Prediction of SRE changes (using Sroogle engine)**

The sequences that were shown to adopt aberrant splicing upon mutation are highlighted in light orange. Positive predictions are marked in green. For an explanation, see material and methods. The statistical comparison of the SRE changes in splicing-affecting and non-affecting samples was counted only from the test-set of sequences (i.e. without the borderline set sequences).

**Table S7. Combined predictions of splicing affection on nine evaluation sequences.**

^a^ Each combined prediction was considered as positive if two (or more) of the three predicted values exceeded the herein proposed cut-off values of the individual tools. The individual values that do not fall into the range of herein proposed cut-off limits are marked in blue. Predictions being in accordance with detected splicing affection are marked in green, the discrepancies are in orange. Py25 = number of pyrimidines in the 25 nucleotides upstream from splice site; ME s.d. = difference between wild type and mutant sequence scores predicted by MaxEnt program; ME p.d. = difference between wild type and mutant sequence percentiles predicted by MaxEnt program; PSSM s.d.: accordingly.

**SUPPLEMENTARY REFERENCES**

S1. Fu Y, Masuda A, Ito M, Shinmi J, Ohno K (2011) AG-dependent 3'-splice sites are predisposed to aberrant splicing due to a mutation at the first nucleotide of an exon. Nucleic Acids Res 39: 4396-4404.

S2. Wang Y, Kanegane H, Wang X, Han X, Zhang Q, et al. (2009) Mutation of the BTK gene and clinical feature of X-linked agammaglobulinemia in mainland China. J Clin Immunol 29: 352-356.

S3. Holzelova E, Vonarbourg C, Stolzenberg MC, Arkwright PD, Selz F, et al. (2004) Autoimmune lymphoproliferative syndrome with somatic Fas mutations. N Engl J Med 351: 1409-1418.

S4. Schwartz S, Hall E, Ast G (2009) SROOGLE: webserver for integrative, user-friendly visualization of splicing signals. Nucleic Acids Res 37: W189-192.

S5. Gaildrat P, Krieger S, Di Giacomo D, Abdat J, Révillion F, et al. (2012) Multiple sequence variants of BRCA2 exon 7 alter splicing regulation. J Med Genet 49: 609-617.

S6. Huang CH, Cheng G, Liu Z, Chen Y, Reid ME, et al. (1999) Molecular basis for Rh(null) syndrome: identification of three new missense mutations in the Rh50 glycoprotein gene. Am J Hematol 62: 25-32.

S7. Aretz S, Uhlhaas S, Sun Y, Pagenstecher C, Mangold E, et al. (2004) Familial adenomatous polyposis: aberrant splicing due to missense or silent mutations in the APC gene. Hum Mutat 24: 370-380.

S8. Will K, Dörk T, Stuhrmann M, Meitinger T, Bertele-Harms R, et al. (1994) A novel exon in the cystic fibrosis transmembrane conductance regulator gene activated by the nonsense mutation E92X in airway epithelial cells of patients with cystic fibrosis. J Clin Invest 93: 1852-1859.

S9. Lualdi S, Pittis MG, Regis S, Parini R, Allegri AE, et al. (2006) Multiple cryptic splice sites can be activated by IDS point mutations generating misspliced transcripts. J Mol Med (Berl) 84: 692-700.

S10. Kanno H, Takizawa T, Miwa S, Fujii H (2004) Molecular basis of Japanese variants of pyrimidine 5'-nucleotidase deficiency. Br J Haematol 126: 265-271.

S11. Shady AA, Colby BR, Cunha LF, Astrin KH, Bishop DF, et al. (2002) Congenital erythropoietic porphyria: identification and expression of eight novel mutations in the uroporphyrinogen III synthase gene. Br J Haematol 117: 980-987.
